# Establishing minimally important differences for cardiac MRI end-points in pulmonary arterial hypertension

**DOI:** 10.1183/13993003.02225-2022

**Published:** 2023-08-03

**Authors:** Samer Alabed, Pankaj Garg, Faisal Alandejani, Krit Dwivedi, Ahmed Maiter, Kavita Karunasaagarar, Smitha Rajaram, Catherine Hill, Steven Thomas, Rebecca Gossling, Michael J. Sharkey, Mahan Salehi, Jim M. Wild, Lisa Watson, Abdul Hameed, Athanasios Charalampopoulos, Haiping Lu, Alex M.K. Rothman, A.A. Roger Thompson, Charlie A. Elliot, Neil Hamilton, Christopher S. Johns, Iain Armstrong, Robin Condliffe, Rob J. van der Geest, Andrew J. Swift, David G. Kiely

**Affiliations:** 1 Department of Infection, Immunity and Cardiovascular Disease, University of Sheffield, Sheffield, UK; 2 Department of Clinical Radiology, Sheffield Teaching Hospitals, Sheffield, UK; 3 INSIGNEO, Institute for *in silico* Medicine, University of Sheffield, Sheffield, UK; 4 Norwich Medical School, University of East Anglia, Norwich, UK; 5 Sheffield Pulmonary Vascular Disease Unit, Royal Hallamshire Hospital, Sheffield, UK; 6 Department of Computer Science, University of Sheffield, Sheffield, UK; 7 Leiden University Medical Center, Leiden, The Netherlands; 8 National Institute for Health and Care Research, Sheffield Biomedical Research Centre, Sheffield, UK; 9 Joint senior authors

## Abstract

**Background:**

Cardiac magnetic resonance (CMR) is the gold standard technique to assess biventricular volumes and function, and is increasingly being considered as an end-point in clinical studies. Currently, with the exception of right ventricular (RV) stroke volume and RV end-diastolic volume, there is only limited data on minimally important differences (MIDs) reported for CMR metrics. Our study aimed to identify MIDs for CMR metrics based on US Food and Drug Administration recommendations for a clinical outcome measure that should reflect how a patient “feels, functions or survives”.

**Methods:**

Consecutive treatment-naïve patients with pulmonary arterial hypertension (PAH) between 2010 and 2022 who had two CMR scans (at baseline prior to treatment and 12 months following treatment) were identified from the ASPIRE registry. All patients were followed up for 1 additional year after the second scan. For both scans, cardiac measurements were obtained from a validated fully automated segmentation tool. The MID in CMR metrics was determined using two distribution-based (0.5sd and minimal detectable change) and two anchor-based (change difference and generalised linear model regression) methods benchmarked to how a patient “feels” (emPHasis-10 quality of life questionnaire), “functions” (incremental shuttle walk test) or “survives” for 1-year mortality to changes in CMR measurements.

**Results:**

254 patients with PAH were included (mean±sd age 53±16 years, 79% female and 66% categorised as intermediate risk based on the 2022 European Society of Cardiology/European Respiratory Society risk score). We identified a 5% absolute increase in RV ejection fraction and a 17 mL decrease in RV end-diastolic or end-systolic volumes as the MIDs for improvement. Conversely, a 5% decrease in RV ejection fraction and a 10 mL increase in RV volumes were associated with worsening.

**Conclusions:**

This study establishes clinically relevant CMR MIDs for how a patient “feels, functions or survives” in response to PAH treatment. These findings provide further support for the use of CMR as a clinically relevant clinical outcome measure and will aid trial size calculations for studies using CMR.

## Extract

In patients with pulmonary arterial hypertension (PAH), symptoms and survival are determined primarily by right ventricular (RV) function. In PAH, a progressive pulmonary vasculopathy results in elevation of mean pulmonary arterial pressure (mPAP) and an increase in RV afterload [1]. With disease progression and chronically elevated mPAP, the right ventricle undergoes remodelling, resulting in either adaptation and maintenance of output [2] or maladaptation, RV failure and consequently reduced survival [3]. Cardiac magnetic resonance (CMR) is the gold standard for assessing the right ventricle and shows potential in the assessment of PAH [4]. Impairments of RV function and associated increases in RV volumes can be detected and quantified by CMR, enabling prediction of clinical worsening and mortality [5] and aiding risk stratification [6]. In addition, CMR is sensitive to improvements in RV function following PAH therapy [7–10] and detects a larger treatment effect than the 6-min walk test (6MWT) [11]. In this context, CMR is an important tool for risk stratification and monitoring of disease and treatment response in PAH [4].

## Tweetable abstract

**Changes in key cardiac MRI metrics reflect changes in how a patient “feels, functions or survives” in pulmonary arterial hypertension** https://bit.ly/3IE21Ow

## Plain language summary

Pulmonary arterial hypertension (PAH) is a disease of the vessels of the lung that causes their narrowing and stiffening. As a result, the heart pumping blood into these diseased lung vessels has to work harder and eventually gets worn out. PAH can affect patients' ability to function in daily activities and impact their quality of life. It also reduces their life expectancy dramatically. Patients are, therefore, often monitored and undergo several investigations to adapt treatment according to their situation. These investigations include a survey of how a patient feels (the emPHasis-10 questionnaire), functions (walking test) and how well the heart is coping with the disease (MRI of the heart). Until now, it is unclear how changes on MRI of the heart reflect changes in how a patient feels and functions. Our study identified patients that had the emPHasis-10 questionnaire, walking test and MRI of the heart at both the time of PAH diagnosis and one year later. This allowed us to compare how the changes in the different tests relate to each other. And because previous research identified thresholds for important changes in the emPHasis-10 questionnaire and the walking tests, we were able to use these tests as a benchmark for changes in the MRI of the heart. Our study identified thresholds for change on heart MRI that might indicate whether a patient has improved or worsened. This finding might have implications for how patients are monitored in clinical practice and future research on PAH treatments.

## Graphical Abstract

**Figure:**
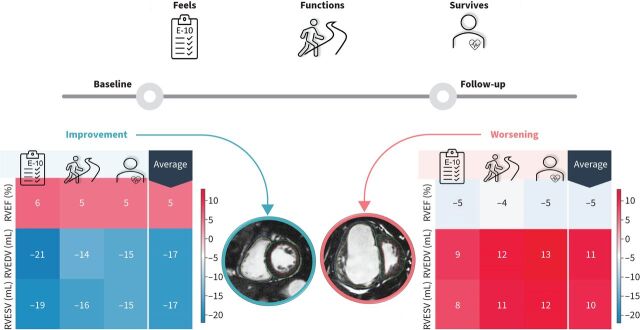
Summary of cardiac magnetic resonance (CMR) absolute change minimally important differences (MIDs) for how a patient “feels” benchmarked to the emPHasis-10 (E-10) quality of life questionnaire, “functions” benchmarked to the incremental shuttle walk test or “survives” benchmarked to 1-year mortality post-follow-up. RVEF: right ventricular ejection fraction; RVEDV: right ventricular end-diastolic volume; RVESV: right ventricular end-systolic volume.

## Introduction

In patients with pulmonary arterial hypertension (PAH), symptoms and survival are determined primarily by right ventricular (RV) function. In PAH, a progressive pulmonary vasculopathy results in elevation of mean pulmonary arterial pressure (mPAP) and an increase in RV afterload [1]. With disease progression and chronically elevated mPAP, the right ventricle undergoes remodelling, resulting in either adaptation and maintenance of output [2] or maladaptation, RV failure and consequently reduced survival [3]. Cardiac magnetic resonance (CMR) is the gold standard for assessing the right ventricle and shows potential in the assessment of PAH [4]. Impairments of RV function and associated increases in RV volumes can be detected and quantified by CMR, enabling prediction of clinical worsening and mortality [5] and aiding risk stratification [6]. In addition, CMR is sensitive to improvements in RV function following PAH therapy [7–10] and detects a larger treatment effect than the 6-min walk test (6MWT) [11]. In this context, CMR is an important tool for risk stratification and monitoring of disease and treatment response in PAH [4].

Phase 4 clinical studies of PAH therapies have recently utilised CMR as a primary end-point in addition to other composite outcomes [9, 10]. Assessing treatment response with CMR necessitates clinically relevant thresholds in order to determine improvement or worsening. However, only RV stroke volume (RVSV) measured on pulmonary artery phase contrast flow imaging has established thresholds [12], while those for volumetric CMR measurements on cine imaging with the exception of RV end-diastolic volume (RVEDV) remain unvalidated [13]. The introduction of automatic volumetric CMR measurements assessing RV changes over time has several advantages. It offers excellent repeatability in scan–rescan assessment and has higher accuracy than manual assessment [14]. In addition, results are generalisable across different centres and magnetic resonance imaging (MRI) systems, allowing standardised comparisons independent of the location of scan [14–16].

The US Food and Drug Administration has highlighted the need to identify clinical outcome measures for PAH therapy trials that reflect how a patient “feels, functions or survives” [17]. To reflect this, we aimed to identify clinically relevant thresholds for change in automatically derived CMR RV and left ventricular (LV) measurements, benchmarking against patient-reported outcome measures (“feels”), exercise testing (“functions”) and mortality (“survives”). Our results should aid the management of patients in the clinic by identifying clinically meaningful changes in key CMR metrics, and aid researchers by informing power calculations and the selection of end-points for clinical studies using CMR.

## Methods

### Study sample

Adult patients with PAH were identified from the ASPIRE (Assessing the Spectrum of Pulmonary hypertension Identified at a REferral centre) registry [18] between January 2010 and January 2022. Diagnosis of PAH was based on mPAP ≥25 mmHg and pulmonary arterial wedge pressure ≤15 mmHg and pulmonary vascular resistance (PVR) ≥3 WU, measured by right heart catheterisation. Patients were eligible for inclusion if they had: 1) baseline CMR prior to starting treatment and within 48 h of PAH diagnosis; 2) follow-up CMR at 12–24 months; and 3) at least 1 year follow-up after the follow-up scan. Patients were excluded if they did not have complete short-axis stack imaging for both baseline and repeat scans. The local ethics committee and institutional review board approved this study (ASPIRE; c06/Q2308/8).

### Imaging procedures

#### MRI protocol

CMR was performed with 1.5 T MRI systems (Signa HDx; GE Healthcare, Chalfont St Giles, UK). Short-axis cine images were acquired using a cardiac-gated multislice balanced steady-state free precession sequence (20 frames per cardiac cycle, section thickness 10 mm, 0 mm inter-section gap, field of view 480 mm, acquisition matrix 256×200, flip angle 60°, bandwidth 125 kHz per pixel, repetition time/echo time (T_R_/T_E_) 3.7/1.6 ms). A stack of images in the short-axis plane was acquired, fully covering both ventricles from base to apex. End-systole was considered to be the smallest cavity area. End-diastole was defined as the first cine phase of the R-wave-triggered acquisition or largest volume. Patients were supine with a surface coil and with retrospective ECG gating.

#### Image analysis

An in-house deep-learning CMR segmentation tool was used to obtain fully automatic measurements [14]. The segmentation tool was trained in a multicentre, multivendor and multipathology dataset, and was previously validated by assessing: 1) accuracy against same-day invasive pulmonary haemodynamics and phase contrast flow imaging; 2) repeatability in a same-day scan–rescan cohort; 3) generalisability in an external testing cohort; and 4) mortality prediction in a large cohort with multiple cardiac and lung pathologies. The automatic contours included trabeculations in the blood pool and were obtained using MASS software (MASS research version 2020; Leiden University Medical Center, Leiden, The Netherlands).

### Clinical parameters

The emPHasis-10 (E-10) questionnaire, a patient-reported outcome measure to assess health-related quality of life in patients with PAH, was completed at baseline and at the time of the follow-up scan from 2014 onwards. Each patient completed 10 questions ranked on a scale of 0 to 5, with a lower score indicating a better quality of life [19]. The incremental shuttle walk test (ISWT) was performed as part of routine patient evaluation according to the standard method [20]. Patients complete a 10 m length keeping in time to an external audible signal. Level 1 consists of three lengths (30 m) and each additional level adds one extra 10 m length to the preceding level. Each level takes 1 min to complete and the test finishes at the end of level 12, a distance of 1020 m. The patient continues until they are too breathless or unable to keep up the required pace. The REVEAL 2.0 and the 2022 European Society of Cardiology (ESC)/European Respiratory Society (ERS) risk scores were calculated from composite clinical parameters [21, 22] and modified to include the ISWT instead of the 6MWT [6, 23]. Mortality data were collected from the electronic records of the National Health Service (NHS) Personal Demographics Service. The NHS automatically updates the mortality records once a death is registered in the UK. All patients were followed up as part of the national service specification for patients with pulmonary hypertension for a minimum of 12 months.

### Statistical analysis

Baseline characteristics are presented as proportion, mean±sd or median (interquartile range (IQR)). For each CMR parameter, both the absolute and relative differences were calculated. The absolute difference was determined by subtracting the baseline measurement from the follow-up measurement, while the relative difference was calculated as the ratio of the absolute difference to the baseline measurement.

We employed four methods to derive the minimally important difference (MID) estimates: two distribution-based and two anchor-based methods (table 1). Initially, Pearson's correlation analysis was used to determine at least a weak correlation (r>0.20) between the change in CMR measurements and the anchor [24, 25]. The difference in CMR parameters and difference in the E-10 or ISWT were regressed onto a scale of 6 units using z-score normalisation to assess the correlation of these changes. Any CMR measurement failing to meet the correlation threshold was subsequently excluded from further MID analysis [24].

**TABLE 1:** Methods employed to calculate the minimally important difference

**Method**	**Type**	**Definition**
**0.5sd**	Distribution-based	Estimated as 0.5 times the sd of the change in CMR measurements within each patient group (improved/worsened).
**Minimal detectable change**	Distribution-based	Calculated based on the sem. The sem was calculated by multiplying the sd of the change in CMR measurements by the square root of (1−reliability coefficient). The formula applied was: MDC=1.96×√2×sem.
**Change difference**	Anchor-based	Difference between the mean change in CMR measurements in patients who had improved or worsened (according to the anchor) and the mean change in stable patients.
**GLM regression**	Anchor-based	Determined by the estimated coefficients for improvement and worsening, derived from a regression analysis using the anchor as a predictor for changes in CMR measurements.

sd: standard deviation; CMR: cardiac magnetic resonance; sem: standard error of measurement; GLM: general linear model.

Anchors for how a patient “feels” and “functions” were determined using a patient-reported outcome measure (E-10) and an assessment of exercise capacity (ISWT), respectively. Patients were considered to have improved, remained stable or worsened between baseline and follow-up, based on a change of 6 points in the E-10 [26, 27] or 47.5 m in the ISWT [28, 29]. For how a patient “survives”, changes in CMR measurements in patients who survived 1 year post-follow-up scan were compared with patients who did not. For the anchor-based method, we derived MID estimates using change difference and regression analysis. The change difference was identified as the difference between the mean change in CMR measurements in patients who had improved or worsened (defined by the anchor) and the mean change in stable patients. The change difference method effectively adjusts the degree of change in the improved or worsened group according to the change observed in the stable group. For the regression analysis, we employed a generalised linear model regression to predict the difference in scores (DScore) between baseline and follow-up CMR measurements, as represented by: DScore=k+βb Xbetter+βw Xworse+βs Xstable The DScore represents the change in CMR parameters and patient status (defined by the anchor as better (Xbetter) or worse (Xworse)) was entered as a dummy variable in the model. The coefficients of better (βb) and worse (βw) in the regression model estimated the incremental difference in scores when patient status transitioned to better or worse compared with stable patients.

In the distribution-based approach, the MID was estimated based on the distribution of CMR measurements within each patient group (improved/worsened). In the first distribution method, the MID was estimated as 0.5 times the standard deviation (0.5sd) of the change in CMR measurements [30]. In the second distribution method, the minimal detectable change (MDC) was calculated using the formula: MDC=1.96×√2×sem, where sem is the standard error of measurement calculated by multiplying the standard deviation of the difference in CMR measurements by the square root of 1 minus its reliability coefficient. Previously published consistency intraclass correlation coefficient values were used as the reliability coefficient of the CMR measurements [14]. Distribution-based methods were employed to facilitate the interpretation of the anchor-based method results. The standard error of measurement describes the variability between the observed and the true measurements. Changes in CMR measurements smaller than the corresponding standard error of measurement are more likely to represent an error of measurement rather than genuine changes [31]. In instances where the anchor-based MIDs were less than the standard error of measurement (*i.e.* indistinguishable from measurement error), the standard error of measurement was utilised as the MID [24].

Statistical analyses were carried out using the lifelines and pingouin Python libraries [32, 33] with a significance threshold of 0.05. Graphs were produced using the Matplotlib library [34] and Prism version 9 (GraphPad, La Jolla, CA, USA).

## Results

**FIGURE 1:**
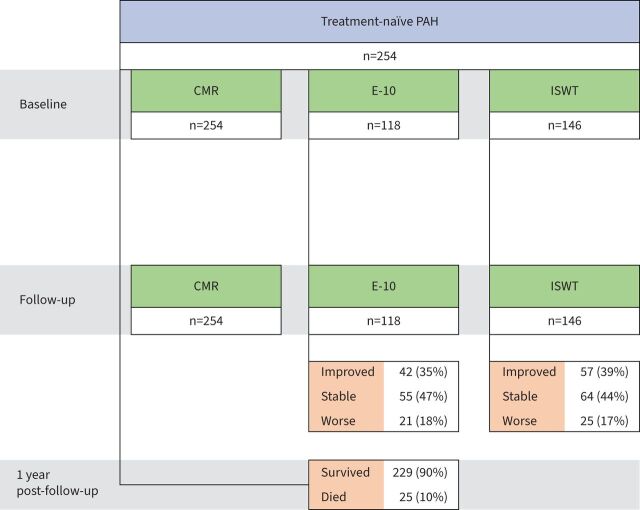
Patient flowchart. First cardiac magnetic resonance (CMR) at baseline→repeat (follow-up) CMR at 12±6 months→1 year post-follow-up. The emPHasis-10 (E-10) health-related quality of life questionnaire and incremental shuttle walk test (ISWT) were performed mostly on the same day and within 2 weeks of CMR. PAH: pulmonary arterial hypertension.

A total of 254 treatment-naïve patients with PAH were included (figure 1). Patients had a mean±sd age of 53±16 years, with 79% female, 83% categorised as World Health Organization (WHO) Functional Class III, and an intermediate risk for mortality of 68% and 66% on REVEAL 2.0 and 2022 ESC/ERS risk models, respectively. 41% had PAH associated with connective tissue disease (PAH-CTD), 37% had idiopathic PAH (IPAH) and 22% had other types of PAH. The median (IQR) mPAP was 50 (41–59) mmHg and PVR was 812 (526–1154) dyn·s·cm^−5^. The mean±sd E-10 score was 31±12 and ISWT walk distance was 207±175 m. Baseline patient characteristics are shown in table 2.

**TABLE 2:** Baseline characteristics (n=254 treatment-naïve patients with pulmonary arterial hypertension (PAH))

**Age (years)**	55 (42–66)
**Female**	201 (79)
**BSA (m^2^)**	1.80±0.35
**PAH subcategory**	
IPAH	94 (37)
CTD	105 (41)
CHD	21 (8)
Portal hypertension	16 (6)
Other PAH	18 (7)
**WHO Functional Class**	
II	10 (4)
III	210 (83)
IV	34 (13)
**REVEAL 2.0 score**	
≤6	11 (4)
7–8	173 (68)
≥9	70 (28)
**2022 ESC/ERS risk**	
Low	18 (7)
Intermediate	169 (66)
High	67 (26)
**RHC parameters**	
mPAP (mmHg)	50 (41–59)
PVR (dyn·s·cm^−5^)	812 (526–1154)
PAWP (mmHg)	10 (8–12)
mRAP (mmHg)	9 (6–14)
CO (L·min^−1^)	4 (3–5)
*S*_vO_2__ (%)	64 (58–70)
Heart rate (beats·min^−1^)	77 (69–89)
**PAH medication**	
PDE5i	
Sildenafil	199 (78)
Tadalafil	20 (8)
ERA	
Ambrisentan	87 (34)
Bosentan	33 (13)
Macitentan	62 (24)
Parenteral prostanoid	43 (17)
Other	11 (4)
**Therapeutic strategy**	
Monotherapy	74 (32)
Dual combination	111 (48)
Triple combination	45 (20)

Data are presented as median (interquartile range), n (%) or mean±sd. BSA: body surface area; IPAH: idiopathic PAH; CHD: congenital heart disease; CTD: connective tissue disease; WHO: World Health Organization; ESC: European Society of Cardiology; ERS: European Respiratory Society; RHC: right heart catheterisation; mPAP: mean pulmonary arterial pressure; PVR: pulmonary vascular resistance; PAWP: pulmonary arterial wedge pressure; mRAP: mean right atrial pressure; CO: cardiac output; *S*_vO_2__: mixed venous oxygen saturation; PDE5i: phosphodiesterase 5 inhibitor; ERA: endothelin receptor antagonist.

The median (IQR) duration between the baseline and repeat (follow-up) scan was 12 (7–16) months. Between the two scans patients were treated with phosphodiesterase 5 inhibitors (86%), endothelin receptor antagonists (72%), parenteral prostanoid (17%) and other medications (4%), with 32% receiving monotherapy, 48% dual combination and 20% triple combination therapy. During follow-up after the repeat scan, 25 out of 254 (10%) patients had died at 12 months and 123 out of 254 (48%) patients died during a median (IQR) period of 5 (3–7) years from baseline.

Table 3 shows the mean CMR measurements at baseline and follow-up for the different patient groups. The absolute differences in RV ejection fraction (RVEF) between baseline and follow-up have been reported in nine studies [8, 10, 11, 35–40] with a total of 321 treatment-naïve patients, and their pooled results compared with the current study are shown in supplementary figure S1 and presented in detail in supplementary table S1. The pooled mean difference in RVEF at 1 year was 6% (95% CI 3–8%) compared with 7% (95% CI 5–9%) in our study.

**TABLE 3:** Changes in cardiac magnetic resonance measurements and clinical parameters: benchmarked to changes in how a patient “feels” (emPHasis-10 (E-10), n=118), “functions” (incremental shuttle walk test (ISWT), n=146) or “survives” (1-year mortality post-follow-up, n=254)

	**Improved**		**Stable**		**Worsened**	
	**Baseline**	**Follow-up**	**Baseline**	**Follow-up**	**Baseline**	**Follow-up**
**E-10**	n=42		n=55		n=21	
E-10 (points)	37±10	21±11	29±12	29±12	24±10	37±7
RVEF (%)	32±12	42±13	34±12	42±10	38±10	43±8
RVEDV (mL)	208±66	182±59	200±78	201±74	174±69	163±57
RVESV (mL)	146±60	110±53	138±68	122±60	112±56	94±40
**ISWT**	n=57		n=64		n=25	
Walk distance (m)	249±181	409±197	197±1501	196±148	259±16	158±142
RVEF (%)	31±10	42±10	34±11	40±10	33±12	38±10
RVEDV (mL)	235±70	216±86	208±79	196±74	187±69	192±70
RVESV (mL)	164±62	128±62	144±68	122±61	129±65	121±55
**Mortality**	Survivors n=229				Non-survivors n=25	
RVEF (%)	34±11	41±11			31±13	36±16
RVEDV (mL)	207±71	195±71			202±79	207±85
RVESV (mL)	141±62	118±56			144±72	139±75

Data are presented as mean±sd, unless otherwise stated. RVEF: right ventricular ejection fraction; RVEDV: right ventricular end-diastolic volume; RVESV: right ventricular end-systolic volume. Measurements were taken at baseline before treatment and at follow-up (12±6 months). Improvement was defined as an increase of at least 47.5 m in the ISWT, a decrease of at least 6 points in the E-10 score and 1 year survival post-follow-up, whereas worsening was defined as a reduction in the ISWT of at least 47.5 m, an increase of 6 points in the E-10 score or mortality. Otherwise, patients were described as stable.

### MIDs for how a patient “feels, functions or survives”

**FIGURE 2:**
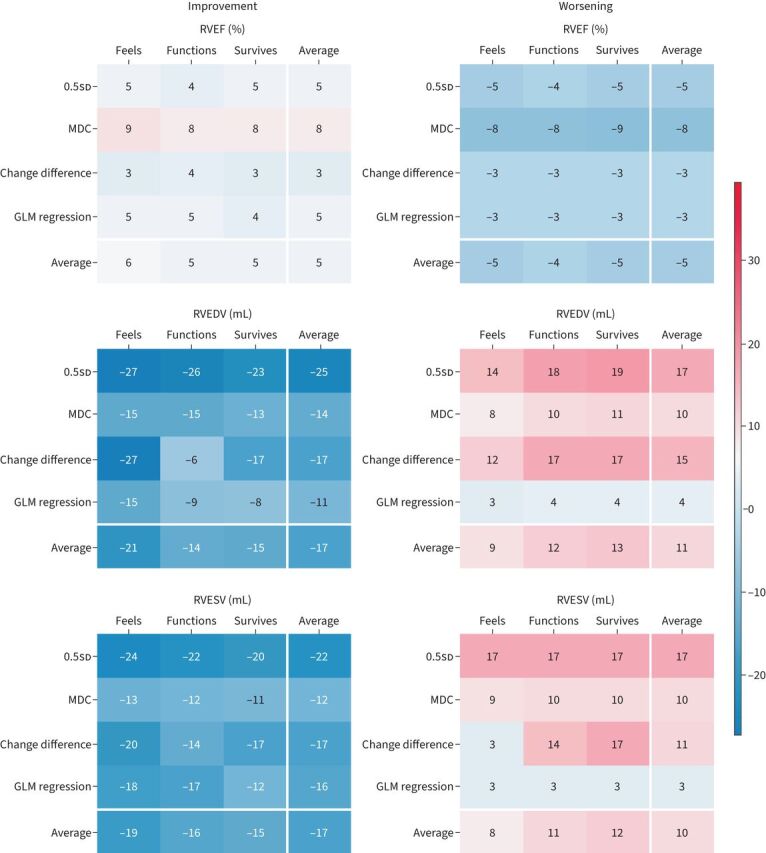
Heatmaps of mean minimally important differences (MIDs) for improvement (left panel) and worsening (right panel) for absolute changes in right ventricular (RV) parameters. The heatmaps display the MIDs calculated using four different assessment methods: 0.5sd, minimal detectable change (MDC), change difference and general linear model (GLM) regression (table 1). The values are colour coded, with blue representing lower MIDs and red representing higher MIDs. The white lines separate the average column and row for each parameter. RVEF: RV ejection fraction; RVEDV: RV end-diastolic volume; RVESV: RV end-systolic volume.

The E-10 and ISWT were performed mostly on the same day as the CMR and within 2 weeks, with a median (IQR) time between the CMR and E-10 of 0 (0–0) days and between the CMR and ISWT of 0 (0–8) days. Paired baseline and follow-up E-10 (n=118) and ISWT (n=146) categorised patients into improved (n=42 (35%) for E-10 and n=57 (39%) for ISWT), stable (n=55 (47%) for E-10 and n=64 (44%) for ISWT) and worsened (n=21 (18%) for E-10 and n=25 (17%) for ISWT) (table 2). The correlation between CMR parameters and the E-10 and ISWT was weak for RV parameters: RVEF (r= −0.25 and r=0.20, respectively), RVEDV (r=0.28 and r= −0.28, respectively) and RV end-systolic volume (RVESV) (r=0.32 and r=0.34, respectively). None of the LV parameters or RVSV (r=0.10 for both the E-10 and ISWT) showed a sufficient correlation with the anchors. The mean MID values for absolute and relative improvement and worsening using the different MID methods are shown in figure 2 and supplementary figure S2. In summary, the overall MID means and range of means across methods for improvement were: 5% (3–9%) for RVEF, −17 mL (−6– −27 mL) for RVEDV and −17 mL (−11– −24 mL) for RVESV. For worsening, the values were −5% (−3– −9%) for RVEF, 11 mL (3–19 mL) for RVEDV and 10 mL (3–17 mL) for RVESV (graphical abstract). The highest relative change, indexed to the baseline value, was observed for RVEF (22% for improvement and −19% for worsening) (supplementary figure S2).

## Discussion

Identifying clinically relevant thresholds for changes in CMR metrics that reflect how a patient “feels, functions or survives” has important implications for patient monitoring and the selection of therapy trial end-points. To the best of our knowledge, this is the first study to compare changes in CMR metrics with health-related quality of life in PAH in addition to measures of function and mortality, and the first to assess clinically relevant MIDs for automatic CMR measurements. MIDs for CMR metrics were identified using various distribution-based and anchor-based methods in an effort to generate reliable estimates [41]. The MIDs obtained using the change in E-10 score, ISWT walk distance and survival as anchors were remarkably consistent across methods and anchors, reinforcing the robustness of the MID estimates. Only RV metrics sufficiently correlated with the anchors to allow for MID calculations.

In this study of 254 patients, we observed a mean absolute difference of 7% in RVEF post-PAH treatment at an average of 1 year follow-up. Our result was comparable to the pooled estimate (6% mean difference in RVEF) identified from nine PAH studies, including 321 PAH patients with pre- and post-treatment RVEF measurements at 9–12 months. Two studies assessed change at 4 months following bosentan therapy: Benza *et al*. [9] showed an absolute increase of 3% in RVEF in 84 patients, and Wilkins *et al*. [42] reported an increase in RVEDV of 6 mL and a decrease of RVESV of 2 mL in 12 patients. van de Veerdonk *et al*. [37] assessed 52 patients at baseline and at 5 years follow-up, and found that a 3% absolute reduction in RVEF was associated with a lower survival rate in patients with decreased PVR. Our study identifies a 5% increase in RVEF and a 17 mL decrease in RVEDV and RVESV as MIDs for improvement, while a 5% decrease in RVEF and a 10 mL increase in RV volumes were associated with worsening.

CMR has been shown to be sensitive to change in response to treatment, with CMR detecting a larger treatment effect than the 6MWT [11]. Bradlow *et al*. [7] previously estimated post-treatment thresholds for RV changes based on four studies with a total of 57 PAH patients. Although they suggested an absolute difference in RVEF of 3% and RV volumes of 10 mL as thresholds, these observations were minimally detectable changes based on the repeatability of the measurement and not benchmarked against clinically relevant outcomes such as changes in quality of life, exercise capacity or mortality. In contrast, in our study we have benchmarked CMR parameters against measures of how a patient “feels, functions or survives”. Until now, the only CMR parameters with a clinically relevant and validated threshold in PAH have been RVSV derived from phase contrast flow imaging [12] and RVEDV measured from trans-axial cine images [13]; RVSV was anchored to a 6MWT, with 10 mL change identified as a threshold for important clinical effect, whereas RVEDV was anchored to a change in WHO Functional Class, with an 11% relative change identified as clinically relevant. In this study, we compared the change in CMR cine imaging to changes in patient-reported outcome measures (E-10 health-related quality of life) and exercise capacity (ISWT walk distance). The E-10 elicits how the patient “feels”, with domains reflecting the burden of breathlessness, fatigue and anxiety on patients with PAH [19], while the ISWT reflects how a patient “functions” by assessing exercise capacity [23, 29, 43]. Both the E-10 and ISWT have established MIDs, can assist in risk stratification of patients and have prognostic value, making them ideal benchmarks for assessing how a patient feels and functions [23, 26]. However, it must be noted that the E-10, ISWT and CMR all measure different components, confirmed by the weak correlation between differences in RV measurements and changes in the E-10 and ISWT, and therefore worsening in quality of life and exercise capacity can occur despite improvements in RV function. Despite being able to demonstrate that CMR metrics can detect MIDs for how a patient “feels, functions or survives”, a composite end-point that includes each of these domains will be superior to using a metric that focuses on a single measure such as cardiac function. Nonetheless, this study does provide further evidence for using CMR as a primary trial end-point in studies of PAH therapies by providing evidence that changes in key CMR metrics do reflect changes in how a patient feels and functions.

While an absolute change in CMR measurements gives an indication of direction, it does not take into account the baseline state of the patient to contextualise the magnitude of change. This may be relevant in patients with more severe disease where relative change is likely to be more sensitive to disease progression by accounting for a patient's pre-treatment baseline and therefore should be considered alongside absolute changes in metrics [44]. In the current study, trabeculations were included in the blood pool. Further work to establish MIDs for CMR measurements excluding trabeculations would be of value and as technology evolves there is a need to develop standardised approaches to CMR measurements of the right ventricle.

### Limitations

The major limitation of any longitudinal retrospective study is the inherent risk of selection bias. Inevitably, patients who survived until follow-up CMR imaging have had a less severe disease course compared with those who died. However, patients having follow-up imaging might have been selected because the treating physician felt they were more at risk of deterioration and required additional monitoring; in our institution (Sheffield Pulmonary Vascular Disease Unit, Royal Hallamshire Hospital, Sheffield, UK), CMR imaging is regularly performed as part of routine follow-up in preference to echocardiography. Furthermore, our findings are based on a single-centre cohort and our mortality prediction thresholds are largely exploratory in nature and should be validated in external cohorts. Given the emergence of prospective studies using CMR imaging as a trial end-point and the increasing use of patient-reported outcome measures, consideration should be given to pooling data from such studies to refine our exploratory thresholds for how a patient “feels, functions or survives”. Larger datasets would also allow for the analysis of potential differences based on disease type (IPAH *versus* PAH-CTD) and sex differences [45, 46]. The E-10 was developed in 2014 and therefore patients included from 2010 to 2014 were only assessed with the ISWT. Finally, we have used the ISWT rather than the 6MWT as a measure of exercise capacity. The ISWT has the benefit over the 6MWT in that it is a maximal test and does not have a ceiling effect [23, 47], and thresholds exist for MIDs [28, 29]; however, data are more limited compared with the 6MWT in patients with PAH. Further study of CMR MIDs benchmarked to other measures of exercise capacity including 6MWT distance is required taking into account established MIDs for the 6MWT [46].

### Conclusions

We have shown that CMR can identify MIDs for how a patient “feels, functions or survives”. In doing so this study provides further evidence that CMR has the characteristics of a clinical outcome measure. In addition, the findings of this study and the description of MIDs will aid trial size calculations for studies using CMR.

## Supplementary material

10.1183/13993003.02225-2022.Supp1 **Please note:** supplementary material is not edited by the Editorial Office, and is uploaded as it has been supplied by the author.
Supplementary material ERJ-02225-2022.Supplement

## Shareable PDF

10.1183/13993003.02225-2022.Shareable1 This one-page PDF can be shared freely online.
Shareable PDF ERJ-02225-2022.Shareable
