# Supplementary material for: Establishing minimally important differences for cardiac MRI end-points in pulmonary arterial hypertension
Source: Eur Respir J. 2023 Aug 3;62(2):2202225. doi: 10.1183/13993003.02225-2022 (PMC10397469; doi:10.1183/13993003.02225-2022)
Supplement: Supplementary file 1 [file ERJ-02225-2022.Supplement.pdf]

**Supplemental Table S1:** Change in RVEF at baseline and follow-up in treatment naive patients with PAH

| STUDY | *No. Patients* | *Follow-up (months)* | *Mean baseline RVEF* | *Mean follow-up RVEF* | *Mean difference [95% CI]* |
|---|---|---|---|---|---|
| **Hassoun 2015** | 23 | 9 | 46 | 57 | 11 [6, 17] |
| **Michelakis 2003** | 3 | 12 | 38 | 45 | 7 [-10, 24] |
| **Peacock 2013** | 71 | 12 | 41 | 46 | 5 [0, 9] |
| **Roeleveld 2004** | 10 | 12 | 34 | 38 | 4 [-7, 15] |
| **Swift 2022*** | 16 | 12 | 32 | 37 | 5 [-3, 13] |
| **Van de Veerdonk 2011** | 76 | 12 | 35 | 36 | 1 [-2, 4] |
| **Van de Veerdonk 2017** | 45 | 12 | 36 | 40 | 4 [-1, 9] |
| **van Wolferen 2006** | 15 | 12 | 33 | 39 | 6 [-9, 21] |
| **Vonk Noordegraaf 2022** | 62 | 12 | 38 | 47 | 10 [5, 15] |
| **Pooled mean** | 321 | 12 | 37 | 43 | 6 [3, 8] |
| **ASPIRE** | 254 | 12 | 34 | 41 | 7 [5, 9] |

* only treatment naive patient included in the analysis

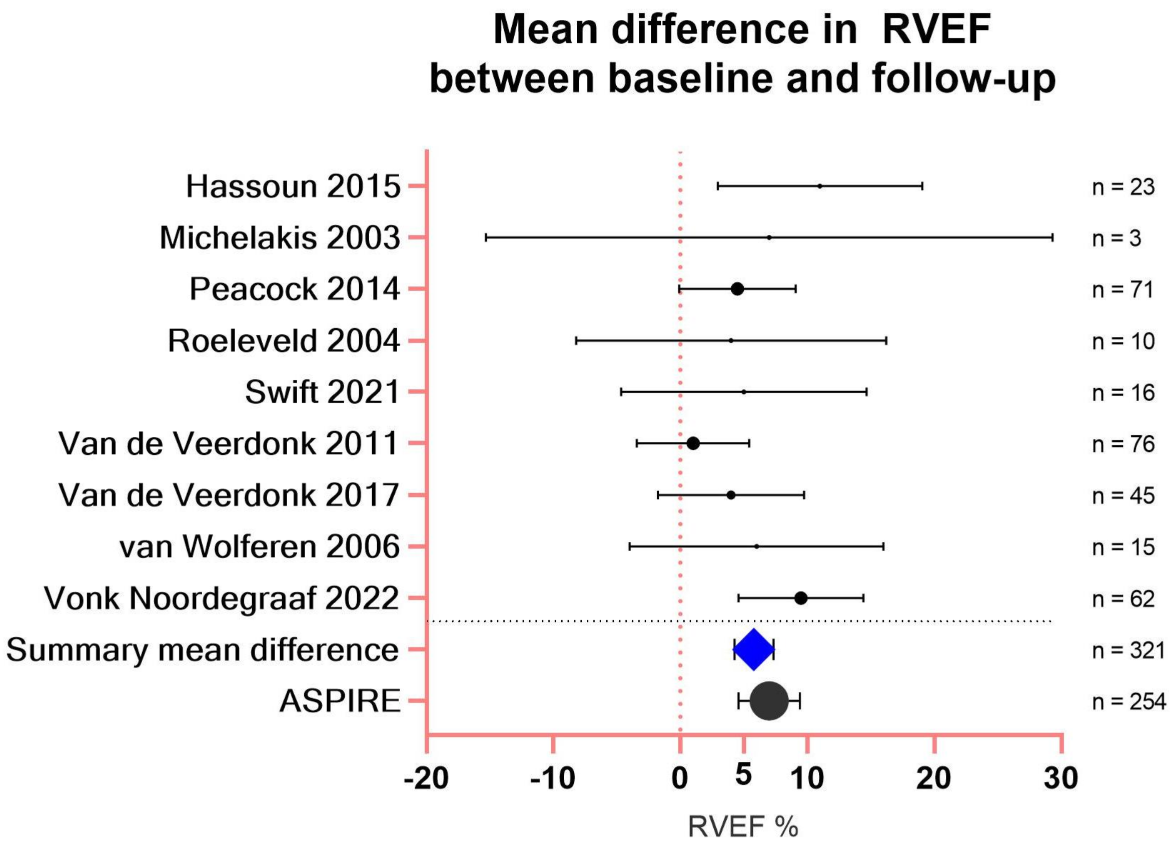


**Supplemental Figure S1:** Pooled summary of mean RVEF differences

Pooled differences of RVEF in treatment-naive PAH patients between baseline and 9-12 months follow-up post-treatment reported in nine studies. The pooled change (blue diamond) in RVEF was 6% (n = 321) compared to 7% in the current ASPIRE study (n= 254).

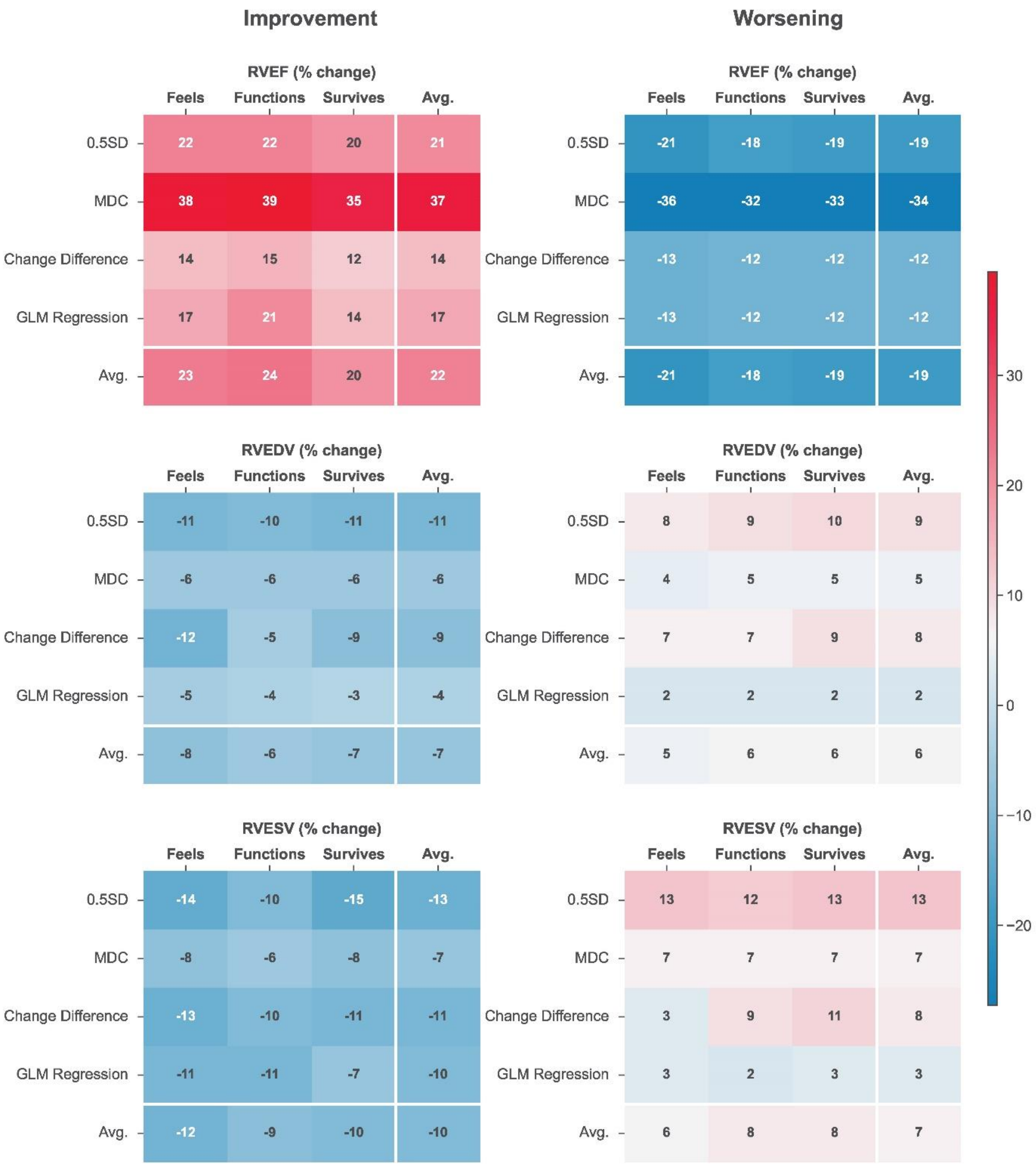


**Supplemental Figure S2:** Heatmap of mean minimal important differences (MID) for improvement (left panel) and worsening (right panel) for relative changes in right ventricular (RV) parameters expressed as change over baseline measurement x100. The heatmaps display the MIDs calculated using four different assessment methods: 0.5 standard deviation (SD), minimal detectable change (MDC), change difference, and

general linear model (GLM) regression. The values are colour-coded, with blue representing lower MIDs and red representing higher MIDs. The bold white lines separate the average column and row for each parameter.

RVEF, right ventricular ejection fraction; RVEDV, right ventricular end-diastolic volume; RVESV, right ventricular end-systolic volume.

## References


Hassoun PM, Zamanian RT, Damico R, Lechtzin N, Khair R, Kolb TM, et al. Ambrisentan and Tadalafil Up-front Combination Therapy in Scleroderma-associated Pulmonary Arterial Hypertension. Am J Respir Crit Care Med. 2015 Nov 1;192(9):1102–10.

Michelakis ED, Tymchak W, Noga M, Webster L, Wu XC, Lien D, et al. Long-term treatment with oral sildenafil is safe and improves functional capacity and hemodynamics in patients with pulmonary arterial hypertension. Circulation. 2003 Oct 28;108(17):2066–9.

Roeleveld RJ, Vonk-Noordegraaf A, Marcus JT, Bronzwaer JGF, Marques KMJ, Postmus PE, et al. Effects of epoprostenol on right ventricular hypertrophy and dilatation in pulmonary hypertension. Chest. 2004 Feb;125(2):572–9.

Peacock AJ., Crawley S., McLure L., et al. Changes in right ventricular function measured by cardiac magnetic resonance imaging in patients receiving pulmonary arterial hypertension-targeted therapy: the EURO-MR study. Circ Cardiovasc Imaging 2014;7(1):107–14.

Swift AJ, Wilson F, Cogliano M, Kendall L, Alandejani F, Alabed S, et al. Repeatability and sensitivity to change of non-invasive end points in PAH: the RESPIRE study [Internet]. Thorax. 2021. p. thoraxjnl – 2020. Available from: http://dx.doi.org/10.1136/thoraxjnl-2020-216078

van de Veerdonk MC, Kind T, Marcus JT, Mauritz GJ, Heymans MW, Bogaard HJ, et al. Progressive right ventricular dysfunction in patients with pulmonary arterial hypertension responding to therapy. J Am Coll Cardiol. 2011 Dec 6;58(24):2511–9.

van de Veerdonk MC., Huis In T Veld AE., Marcus JT., et al. Upfront combination therapy reduces right ventricular volumes in pulmonary arterial hypertension. Eur Respir J 2017;49(6). Doi: 10.1183/13993003.00007-2017.

Vonk Noordegraaf A, Channick R, Cottreel E, Kiely DG, Marcus JT, Martin N, et al. The REPAIR Study: Effects of Macitentan on RV Structure and Function in Pulmonary Arterial Hypertension. JACC Cardiovasc Imaging. 2022 Feb;15(2):240–53.

van Wolferen SA, Boonstra A, Marcus JT, Marques KMJ, Bronzwaer JGF, Postmus PE, et al. Right ventricular reverse remodelling after sildenafil in pulmonary arterial hypertension. Heart. 2006 Dec;92(12):1860–1.
