# Supplementary material for: Establishing minimally important differences for cardiac MRI end-points in pulmonary arterial hypertension
Source: Eur Respir J. 2023 Aug 3;62(2):2202225. doi: 10.1183/13993003.02225-2022 (PMC10397469; doi:10.1183/13993003.02225-2022)
Supplement: Supplementary file 2 [file ERJ-02225-2022.Shareable.pdf]

EUROPEAN RESPIRATORY JOURNAL
ORIGINAL RESEARCH ARTICLE
S. ALABED ET AL.

SHAREABLE PDF

Establishing minimally important differences for cardiac MRI end-points in pulmonary arterial hypertension

Samer Alabed, Pankaj Garg, Faisal Alandejani, Krit Dwivedi, Ahmed Maiter, Kavita Karunasaagarar, Smitha Rajaram, Catherine Hill, Steven Thomas, Rebecca Gossling, Michael J. Sharkey, Mahan Salehi, Jim M. Wild, Lisa Watson, Abdul Hameed, Athanasios Charalampopoulos, Haiping Lu, Alex M.K. Rothman, A.A. Roger Thompson, Charlie A. Elliot, Neil Hamilton, Christopher S. Johns, Iain Armstrong, Robin Condliffe, Rob J. van der Geest, Andrew J. Swift and David G. Kiely

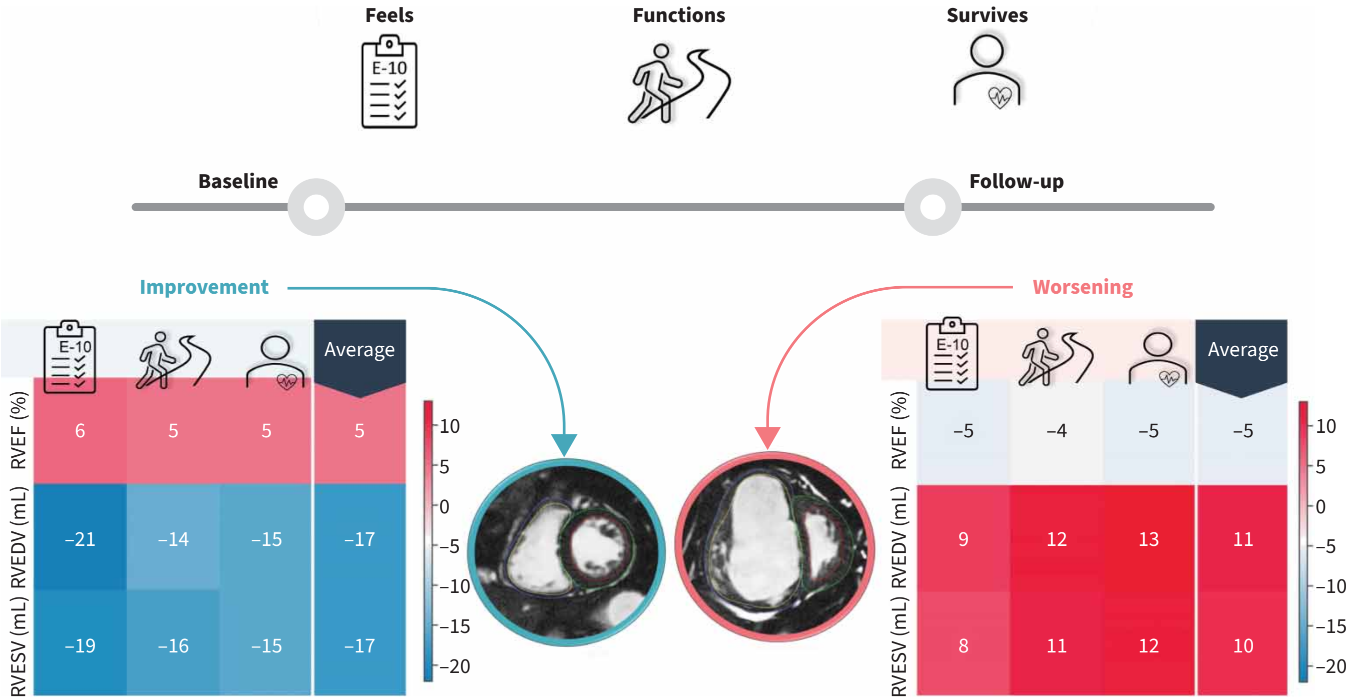

GRAPHICAL ABSTRACT Summary of cardiac magnetic resonance (CMR) absolute change minimally important differences (MIDs) for how a patient "feels" benchmarked to the emPHasis-10 (E-10) quality of life questionnaire, "functions" benchmarked to the incremental shuttle walk test or "survives" benchmarked to 1-year mortality post-follow-up. RVEF: right ventricular ejection fraction; RVEDV: right ventricular end-diastolic volume; RVESV: right ventricular end-systolic volume.

EUROPEAN RESPIRATORY JOURNAL
ORIGINAL RESEARCH ARTICLE
S. ALABED ET AL.

SHAREABLE PDF

Establishing minimally important differences for cardiac MRI end-points in pulmonary arterial hypertension

Samer Alabed [1,2,3], Pankaj Garg[4], Faisal Alandejani [1], Krit Dwivedi[1,2], Ahmed Maiter[1,2], Kavita Karunasaagarar[1,2], Smitha Rajaram[1,2], Catherine Hill[2], Steven Thomas[1,2], Rebecca Gossling[1], Michael J. Sharkey [1], Mahan Salehi[1], Jim M. Wild[1,3], Lisa Watson[5], Abdul Hameed [5], Athanasios Charalampopoulos[5], Haiping Lu[3,6], Alex M.K. Rothman[1], A.A. Roger Thompson [1,5], Charlie A. Elliot[1,5], Neil Hamilton[1,5], Christopher S. Johns[1,2], Iain Armstrong[1,5], Robin Condliffe [1,5], Rob J. van der Geest[7], Andrew J. Swift[1,2,3,8,9] and David G. Kiely [1,3,5,8,9]

[1]Department of Infection, Immunity and Cardiovascular Disease, University of Sheffield, Sheffield, UK. [2]Department of Clinical Radiology, Sheffield Teaching Hospitals, Sheffield, UK. [3]INSIGNEO, Institute for *in silico* Medicine, University of Sheffield, Sheffield, UK. [4]Norwich Medical School, University of East Anglia, Norwich, UK. [5]Sheffield Pulmonary Vascular Disease Unit, Royal Hallamshire Hospital, Sheffield, UK. [6]Department of Computer Science, University of Sheffield, Sheffield, UK. [7]Leiden University Medical Center, Leiden, The Netherlands. [8]National Institute for Health and Care Research, Sheffield Biomedical Research Centre, Sheffield, UK. [9]Joint senior authors.

Corresponding author: Samer Alabed (s.alabed@nhs.net)

Check for updates

Shareable abstract (@ERSpublications)
Changes in key cardiac MRI metrics reflect changes in how a patient "feels, functions or survives" in pulmonary arterial hypertension https://bit.ly/3IE21Ow

Cite this article as: Alabed S, Garg P, Alandejani F, *et al*. Establishing minimally important differences for cardiac MRI end-points in pulmonary arterial hypertension. *Eur Respir J* 2023; 62: 2202225 [DOI: 10.1183/13993003.02225-2022].

This single-page version can be shared freely online.

Copyright ©The authors 2023.

This version is distributed under the terms of the Creative Commons Attribution Licence 4.0.

Received: 20 Nov 2022
Accepted: 23 May 2023

Abstract

Background Cardiac magnetic resonance (CMR) is the gold standard technique to assess biventricular volumes and function, and is increasingly being considered as an end-point in clinical studies. Currently, with the exception of right ventricular (RV) stroke volume and RV end-diastolic volume, there is only limited data on minimally important differences (MIDs) reported for CMR metrics. Our study aimed to identify MIDs for CMR metrics based on US Food and Drug Administration recommendations for a clinical outcome measure that should reflect how a patient "feels, functions or survives".

Methods Consecutive treatment-naïve patients with pulmonary arterial hypertension (PAH) between 2010 and 2022 who had two CMR scans (at baseline prior to treatment and 12 months following treatment) were identified from the ASPIRE registry. All patients were followed up for 1 additional year after the second scan. For both scans, cardiac measurements were obtained from a validated fully automated segmentation tool. The MID in CMR metrics was determined using two distribution-based (0.5SD and minimal detectable change) and two anchor-based (change difference and generalised linear model regression) methods benchmarked to how a patient "feels" (emPHasis-10 quality of life questionnaire), "functions" (incremental shuttle walk test) or "survives" for 1-year mortality to changes in CMR measurements.

Results 254 patients with PAH were included (mean±SD age 53±16 years, 79% female and 66% categorised as intermediate risk based on the 2022 European Society of Cardiology/European Respiratory Society risk score). We identified a 5% absolute increase in RV ejection fraction and a 17 mL decrease in RV end-diastolic or end-systolic volumes as the MIDs for improvement. Conversely, a 5% decrease in RV ejection fraction and a 10 mL increase in RV volumes were associated with worsening.

Conclusions This study establishes clinically relevant CMR MIDs for how a patient "feels, functions or survives" in response to PAH treatment. These findings provide further support for the use of CMR as a clinically relevant clinical outcome measure and will aid trial size calculations for studies using CMR.
